# Objectively measured crime and active transportation among 10–13 year olds

**DOI:** 10.1016/j.pmedr.2018.11.010

**Published:** 2018-11-20

**Authors:** Mijal Vonderwalde, Justyna Cox, Gillian C. Williams, Michael M. Borghese, Ian Janssen

**Affiliations:** a School of Kinesiology and Health Studies, Queen's University, Kingston, ON, Canada; b Department of Public Health Sciences, Queen's University, Kingston, ON, Canada

**Keywords:** Child, Adolescent, Physical activity, Walking, Crime

## Abstract

This study examined the temporal relationship between objective measures of neighborhood crime and active transportation among children. A sample of 387 children aged 10–13 years from Kingston, Canada were studied between January 2015 and December 2016. Active transportation was measured over 7 days using Geographic Information System loggers. The number of crimes per capita were measured within a 1 km distance of participants' homes for the 24-month period prior to when their active transportation was measured. Surprisingly, children living in neighborhoods in the highest neighborhood crime rate quartile engaged in significantly more active transportation than children living in neighborhoods in the lowest neighborhood crime rate quartile (16.4 versus 10.2 min/day, p < 0.05). This relationship persisted after adjustment for several individual, family, and environmental covariates.

## 1 Introduction

Public health guidelines recommend that school-aged children accumulate 60 min/day of moderate-to-vigorous physical activity (World Health Organization, 2010). Most young people do not achieve this guideline (Hallal et al., 2012). Active transportation is one way that children can accumulate moderate-to-vigorous physical activity; however, many children engage in little or no active transportation (Tremblay et al., 2016). In fact, for 69% of the countries that participated in the 2016 global matrix on children's physical activity, the grade for active transportation was a C or lower, which indicates that <60% of children in these countries regularly used active transportation (Tremblay et al., 2016).

Crime is a potential determinant of active transportation (Carver et al., 2005; Kerr et al., 2006; Vanwolleghem et al., 2016; Cutumisu et al., 2014). It has been theorized that parents living in unsafe neighborhoods restrict their child's independent mobility and active transportation to protect them from crime and other dangers. (Carver et al., 2008) This theory is supported by empirical evidence from several studies which found that perceptions of crime are negatively associated with children's active transportation (Carver et al., 2005; Kerr et al., 2006; Vanwolleghem et al., 2016; Cutumisu et al., 2014). Although these studies have made important contributions, their findings should be interpreted with caution because they mostly used cross-sectional designs and because they relied on subjective measures of active transportation, which are biased (Adamo et al., 2009). Furthermore, these studies examined perceptions of crime, which are important, but at the same time poorly correlate with actual crime rates (Janssen, 2014). Thus, it is unclear if active transportation is associated with crime rates in a similar manner to which it is associated with perceptions of crime.

Two studies have explored whether objectively measured crime is related to accelerometer-derived measures of moderate-to-vigorous physical activity, some of which would have been accrued through active transportation (Kneeshaw-Price et al., 2015; Jago et al., 2006). One of these studies found that neighborhood crime rate was negatively associated with moderate-to-vigorous physical activity among 145 children aged 6–11 years from San Diego, California (Kneeshaw-Price et al., 2015). The other found that neighborhood crime rate was not associated with moderate-to-vigorous physical activity among 210 boys aged 10–14 years from Houston, Texas (Jago et al., 2006). Collectively, the lack of longitudinal studies, lack of studies using objective measures of active transportation, and inconsistency in the literature highlight the need for additional research. Therefore, the purpose of this short communication was to examine the temporal relationship between objective measures of neighborhood crime and objectively measured active transportation among 10–13 year olds.

## 2 Methods

### 2.1 Participants

Data from this study are from the Active Play Study, a study of 458 children aged 10–13 years from Kingston, Ontario, Canada. Kingston is a mid-sized city with a population of 123,798 people. Consent was obtained from participants and a parent/guardian. Ethics approval was obtained from the General Research Ethics Board of Queen's University. Recruitment methods ensured proportional representation across age, gender, season of participation, and Kingston's 12 electoral districts. Data on active transportation for each participant was collected over 7 consecutive days at some point between January 2015 and December 2016. Their neighborhood crime data were abstracted for the 24-month period that immediately preceded when their active transportation was measured (e.g., for a participant who had their active transportation measured in December 2016, their neighborhood crime data were abstracted for December 2014 to November 2016). Thus, the neighborhood crime measures temporally preceded the active transportation measures.

### 2.2 Neighborhood crime

Crime data for Kingston was obtained from www.CrimeReports.com. This database contains the date, address, and type of crimes reported to the Kingston police force, including crimes against persons (assault and sexual assault) and crimes against property (robbery, break and enter, theft, and vehicle theft). The address of each crime was geocoded using ArcGIS software version 10.4 (ESRI, Redlands, California, USA). Based on existing precedence in the Canadian literature (Seliske et al., 2012), a 1 km road network buffer surrounding each participant's home was created to define their home neighborhood. We then determined the number of total crimes, crimes against persons, and crimes against property that occurred within each participant's buffer for the 24-month period that immediately preceded the 7 day period during that their active transportation was measured.

PCensus 10 for MapPoint software (Tetrad, Ferndale, WA) was used to determine the population within each neighborhood buffer based on the 2011 Canadian Census of Population. Based on the number of crimes and population we calculated the annual crime rates per 10,000 persons for all crimes, crimes against person, and crimes against property. Participants were grouped into quartiles based on the crime rate in their neighborhood.

### 2.3 Active transportation

Participants wore a Garmin Forerunner 220 Global Positioning System (GPS) watch (Garmin Ltd., Schaffhausen, Switzerland) for 7 consecutive days. The watch continuously recorded their geographic position during waking hours. GPS data from the watch were imported into the Personal Activity and Location Measurement System (PALMS) software. PALMS has been validated as a method for estimating time spent in different travel modes (e.g., vehicle travel, walking, bicycling) (Carlson et al., 2015). It uses an algorithm to identified trips based on sequential GPS points that spanned ≥100 m with a speed ≥1 km/h lasting ≥3 min. PALMS allowed for pauses of ≤3 min to account for brief stops (e.g., red traffic light). The modality of each trip was classified as passive (i.e., vehicle at ≥25 km/h) or active (i.e., walking or bicycle at 1–24.99 km/h).

We used Google Fusion Tables software (Google, Mountain View, California, USA) to visually inspect each trip identified by PALMS to identify false positives. False positives are those trips identified by PALMS that reflect other movements such as outdoor play or sports. A total of 6595 trips that were identified that occurred solely within a specific location (e.g., the school yard at recess) were considered false positives and were manually deleted.

Data were then imported into SAS 9.4 statistical software (SAS Institute, Cary, North Carolina, USA) to calculate the average minutes/day of active transportation. During this process we deleted data from days with <10 h of GPS data and participants with <4 days with 10 h of GPS data. Although common rules do not exist for the processing of GPS data, this deletion step is consistent with processing commonly done with accelerometer measures of physical activity (Colley et al., 2010). Seventy (15%) participants were removed during this step.

### 2.4 Covariates

Child-level covariates were biological sex, age, and race. Family-level covariates were the number of siblings in the household, single or dual parent household, family income, and parental education. Environmental-level covariates were the season of data collection, a walkability index that was based on measures of street connectivity (length of roads, intersection density, average block length, connected node ratio), proximity to destinations (Walk Score®, distance to school, population density), and pedestrian safety from traffic (length of sidewalks, traffic volume, traffic speed, density of traffic calming measures) (Williams et al., 2018).

### 2.5 Statistical analysis

Data were analyzed using SAS version 9.4. Because 11% of participants did not engage in any active transportation, a two-part modelling strategy was used to estimate group averages for active transportation. In the first part, logistic regression was used to model the probability of the presence of any active transportation according to crime. In the second part, a generalized linear model was used to model the relationship between crime and active transportation minutes in those who engaged in active transportation. All models were adjusted for age, sex, and season. Other covariates were entered using backwards selection methods and were retained based on a significance level of p < 0.1. Average daily minutes of active transportation was calculated by multiplying the estimates from the two models together. ANOVA with Bonferroni post hoc comparisons were used to compare active transportation across groups.

## 3 Results

Descriptive characteristics of the 367 participants included in the final analyses are in Table 1. Approximately half were female and there were comparable numbers of 10, 11, 12, and 13 year olds. >85% were white, came from dual parent households, and lived with at least one sibling. On average, participants spent 11.8 min/day (95% CI: 8.6, 16.2) engaged in active transportation.

**Table 1:** Participant characteristics (Kingston, Canada; Jan 2015-Dec 2016).

Characteristic	N	%
Sex		
Male	185	50.4
Female	182	49.6
Age		
10 years	89	24.3
11 years	89	24.3
12 years	98	26.7
13 years	91	24.8
Race		
White	316	86.1
Other	51	13.9
Season of participation		
Winter	96	26.2
Spring	92	25.1
Fall	87	23.7
Summer	92	25.1
Number of parents in household		
Dual parent	314	85.6
Single parent	50	13.6
No response	3	0.8
Number of siblings in household		
0	46	12.5
1	190	51.8
2	91	24.8
3+	40	10.9
Family income ($ CDN per year)		
≤50,000	55	15.0
50,001–100,000	108	29.4
>100,000	165	45.0
No response	39	10.6
Parental education		
High school or less	31	8.5
2-Year college	108	29.4
4-Year college/university	228	62.1

A breakdown of active transportation according to neighborhood crime, after adjustment for relevant covariates (age, sex, season, number of parents and siblings in the household, and walkability index), is in Table 2. Children in the highest neighborhood crime quartile had significantly higher active transportation levels than children in quartiles 1 and 3 (p < 0.05). Active transportation levels did not differ significantly across crimes against property quartiles; however, they were higher in quartile 4 than in quartile 1 when the quartiles were determined based on crimes against persons (p < 0.05). The patterns of findings were similar when analyses were stratified by sex, age, and season of study (data not shown). There were no significant interactions between crime rates and these factors.

**Table 2:** Average minutes/day of active transportation according to neighborhood crime rate quartile in the total sample for all crimes, crimes against property, and crimes against persons (Kingston, Canada; Jan 2015-Dec 2016).

Characteristic	Neighborhood crime rate quartile				P trend
	1	2	3	4	
All crimes^c^	10.2 (7.6, 13.9)	10.6 (7.6, 15.1)	9.7 (7.1, 13.3)	16.4 (11.6, 23.2)^a^, ^b^	0.029
Crimes against property^d^	9.8 (7.2, 13.4)	11.3 (8.3, 15.4)	10.3 (7.4, 14.7)	15.5 (10.9, 22.2)	0.132
Crimes against persons^e^	10.7 (7.8, 15.0)	10.4 (7.6, 14.2)	9.1 (6.6, 12.5)	16.6 (11.8, 23.5)^a^	0.064

Data presented as mean minutes/day (95% confidence interval). Values adjusted for age, sex, season, number of parents and siblings in the household, and walkability index.

a Significantly different vs. quartile 1.

b Significantly different vs. quartile 3.

c Crime rate (crimes per year per 10,000 persons) ranges were as follows: quartile 1 = 0 to 36.8, quartile 2 = 36.9 to 60.9, quartile 3 = 61.3 to 156.4, quartile 4 = 159.0 to 988.0.

d Crime rate (crimes per year per 10,000 persons) ranges were as follows: quartile 1 = 0 to 28.6, quartile 2 = 28.8 to 49.8, quartile 3 = 50.3 to 115.4, quartile 4 = 116.1 to 793.1.

e Crime rate (crimes per year per 10,000 persons) ranges were as follows: quartile 1 = 0 to 5.3, quartile 2 = 5.4 to 11.1, quartile 3 = 11.2 to 30.5, quartile 4 = 31.0 to 194.2.

## 4 Discussion

We examined the temporal relationship between objective measures of neighborhood crime and objectively measured active transportation among 10–13 year olds from Kingston, Canada. Surprisingly, we found that children living in neighborhoods with the highest crimes rates engaged in the most active transportation.

In contrast to our findings, previous studies have, in general, reported that perceptions of crime risk are negatively associated with subjectively measured active transportation among children. (Carver et al., 2005; Kerr et al., 2006; Vanwolleghem et al., 2016; Cutumisu et al., 2014) Previous research has also shown that perceptions of crime risk are only modestly correlated with actual crime rates (Janssen, 2014). Taken together, these findings suggest that perceptions of crime is the more relevant determinant of human behavior and a more important target for intervention. The discrepancy between our findings and earlier studies could also reflect that the crime environment in Kingston, the city where our study was conducted, is different from the crime environment in the cities where the earlier studies were conducted. Specifically, the crime rates in Kingston are lower than they are in most cities. In fact, Kingston had the second lowest violent crime rate of all municipalities in Canada with a population > 100,000 (Keighley, 2017). Thus, it is likely that Kingston neighborhoods with the highest crime rates would not be perceived as being as unsafe for children to engage in active transportation by comparison to neighborhoods with the highest crimes in most other cities. The unexpected association between crime rates and active transportation in our study might also reflect residual confounding. Kingston neighborhoods with the highest crime rates are located in the poorest areas of the city. Although we controlled for household income in the statistical analyses, we did not measure or control for household vehicle ownership, which was likely lower in participants living in these poorer neighborhoods.

Our findings both surprised and encouraged us because they suggest that in cities with low crime rates, such as Kingston, children from socioeconomically disadvantaged neighborhoods are not at a disadvantage when it comes to active transportation. Thus, interventions aimed at increasing active transportation levels in such cities could focus on other determinants such as perceptions of crime and walkability. A recent study based on the same sample of children included in this paper found that children living in the most walkable neighborhoods engaged in three times more active transportation than did children living in the least walkable neighborhoods (Williams et al., 2018).

Our study was not void of limitations. The study sample was limited to 10–13 year olds living in Kingston, a mid-sized city with a low crime rate. Active transportation levels differ among children of different ages and among children living in urban and rural environments (Larouche et al., 2014). Further, the present study only assessed active transportation over the course of one week, and these data may not be reflective of all participants' habitual active transportation levels (Baranowski et al., 2008). Finally, although active travel was determined using GPS data in an objective manner using a validated algorithm (Carlson et al., 2015), some of the vehicle trips may have been misclassified as active transportation.

## 5 Conclusion

The results of this longitudinal study indicate that objectively measured neighborhood crime rates did not have a negative influence on children's active transportation. Future studies should consider whether the relationship between crime and active transportation is limited to larger cities with higher crime rates.
